# Polysaccharide-Based Composite Systems in Bone Tissue Engineering: A Review

**DOI:** 10.3390/ma17174220

**Published:** 2024-08-27

**Authors:** Karina Niziołek, Dagmara Słota, Agnieszka Sobczak-Kupiec

**Affiliations:** 1 Cracow University of Technology, CUT Doctoral School, Faculty of Materials Engineering and Physics, Department of Materials Science, 37 Jana Pawła II Av., 31-864 Krakow, Poland; dagmara.slota@pk.edu.pl; 2 Cracow University of Technology, Faculty of Materials Engineering and Physics, Department of Materials Science, 37 Jana Pawła II Av., 31-864 Krakow, Poland; agnieszka.sobczak-kupiec@pk.edu.pl

**Keywords:** biomaterials, polysaccharides, biopolymers, composites, hydroxyapatite

## Abstract

In recent years, a growing demand for biomaterials has been observed, particularly for applications in bone regenerative medicine. Bone tissue engineering (BTE) aims to develop innovative materials and strategies for repairing and regenerating bone defects and injuries. Polysaccharides, due to their biocompatibility, biodegradability as well as bioactivity, have emerged as promising candidates for scaffolds or composite systems in BTE. Polymers combined with bioactive ceramics can support osteointegration. Calcium phosphate (CaP) ceramics can be a broad choice as an inorganic phase that stimulates the formation of new apatite layers. This review provides a comprehensive analysis of composite systems based on selected polysaccharides used in bone tissue engineering, highlighting their synthesis, properties and applications. Moreover, the applicability of the produced biocomposites has been analyzed, as well as new trends in modifying biomaterials and endowing them with new functionalizations. The effects of these composites on the mechanical properties, biocompatibility and osteoconductivity were critically analyzed. This article summarizes the latest manufacturing methods as well as new developments in polysaccharide-based biomaterials for bone and cartilage regeneration applications.

## 1. Introduction

The field of bone tissue engineering (BTE) has seen significant progress in recent years, driven by the growing demand for the effective treatment of bone defects and diseases. Among the various strategies being explored, the use of biomaterials has become a key aspect of the development of scaffolds to aid bone regeneration [1]. According to the Market And Market report, the global biomaterials market was valued at USD 45.2 billion in 2024. Moreover, the market is estimated to reach USD 64.2 billion by 2029 at a compound annual growth rate (CAGR) of 7.3%. The apparent growth is due to increased funding for regenerative medicine, particularly personalized therapies, as well as growing demand for biomaterials in wound-healing applications. North America is the largest leader in market share for biomaterials, with Asia-Pacific regions accounting for the highest growth rate [2]. In particular, polysaccharide-based biomaterials have gained considerable attention due to their biocompatibility, biodegradability and ability to mimic the natural extracellular matrix (ECM) of bone tissue [3,4].

Polysaccharides (PSAs) are natural, renewable biomacromolecules that are found in many living organisms. They contain sugar units—saccharose—in their structure. Moreover, these are materials that contain a high content of functional groups in their structure, such as amino (e.g., in chitosan), carboxyl (e.g., in hyaluronic acid) or hydroxyl groups. PSAs can be an excellent alternative to synthetic polymers. These biopolymers are non-toxic, biodegradable and relatively inexpensive. Polysaccharides, which include materials such as pullulan, chitosan, dextran, and hyaluronic acid, are used to produce scaffolds. Their inherent properties, such as their ability to form hydrogels and membranes, make them suitable for a variety of biomedical applications. Moreover, these materials can be chemically modified to increase their mechanical strength, bioactivity, and ability to interact with cells, further expanding their range of applications.

In order to conduct the literature review presented here, a search of the electronic databases Science Direct, Google Scholar and Wiley, for works published mainly between 2008 and 2024, was performed. The literature review considered the criterion of polysaccharide-based biomaterials in combination with calcium phosphate ceramics. Subsequently, this article was written on the basis of an analysis of 160 scientific research studies, which were cited in the latest updated publications. The largest number of literature reports on this topic are from 2020 to 2024. The databases were searched based on keywords such as “biomaterials”, “polysaccharides”, “biopolymers”, “composites” and “hydroxyapatite”.

This review aims to provide an overview of current information on biomaterials in bone tissue regeneration based on selected PSAs. The basic properties of selected polysaccharides, their use in creating composite systems, their advantages and limitations, as well as recent advances in their applications will be discussed. The review presented here is limited to studies of calcium phosphate ceramic composites due to the fact that no other literature reviews were found on the topic under study. By exploring the potential of polysaccharide-based scaffolds, this review aims to highlight their importance to the future of bone tissue engineering and regenerative medicine.

## 2. Biomedical Applications of Polysaccharide-Based Biomaterials

**Figure 1:**
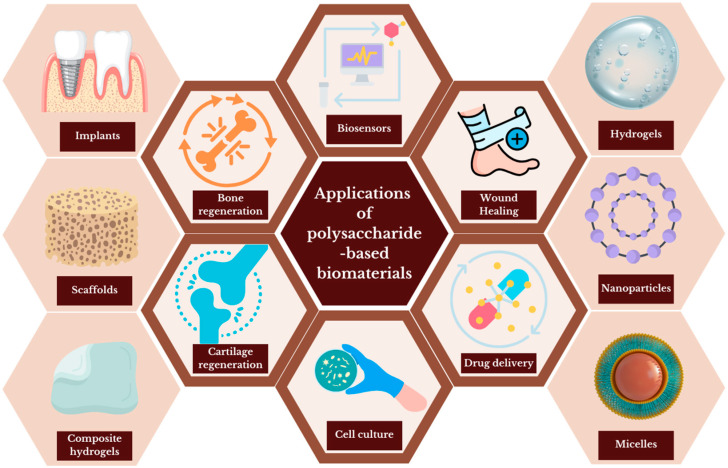
Applications of polysaccharide-based biomaterials in regenerative medicine.

Polysaccharides are of great interest to researchers, particularly in relation to medical applications. Due to their properties, such as biocompatibility or nontoxicity, some polysaccharides have been recognized by the Food and Drug Administration (FDA) as generally safe. Moreover, PSAs can be modified to improve the solubility, stability or viscosity. They exhibit a wide range of applications, as illustrated in Figure 1. PSAs used as potency for biomedical applications include chitosan, hyaluronic acid, pullulan, arabinoxylans, inulin, dextran, chondroitin sulfate and heparin. The advantages and disadvantages of selected polysaccharides are listed in Table 1.

Polysaccharide-based biomaterials are most commonly used in bone and cartilage regeneration. Three main trends can be distinguished in the research: composite biomaterials, hydrogels, scaffolds and 3D-printed implants using various techniques.

Hydrogel composite biomaterials and scaffolds for bone and cartilage regeneration play a key role in modern regenerative medicine. Composite hydrogels, due to their specific structure and the mechanical properties of ceramics, are ideal materials for creating an environment conducive to cell proliferation and tissue regeneration. They consist of a polymer matrix that can be modified to achieve the desired characteristics, such as elasticity, porosity and the ability for controlled drug release. The bioactivity of the created materials is imparted by bioceramics [5]. Scaffolds for osteochondral tissue regeneration, manufactured from composite biomaterials, are designed not only to promote cell growth but also to restore the structure and function of damaged tissues. Thanks to advanced techniques such as 3D printing, it is possible to precisely design and create scaffolds with complex architecture that mimic natural tissue structures. In addition, the integration of bioactive molecules into these structures promotes healing and regeneration [6].

Polysaccharide-based drug delivery systems (DDSs) are an innovative approach in the field of pharmacology and regenerative medicine due to their unique biocompatible and biodegradable properties. Polysaccharides, such as chitosan or hyaluronic acid, are used to create matrices that can control drug release, protect active substances from degradation and enhance their bioavailability. These matrices can be designed in various forms, such as microspheres, nanoparticles, films or hydrogels, tailored to specific therapeutic needs [7,8]. With the possibility of chemical modifications, PSAs can be functionalized to increase the specificity of drug delivery to specific cells or tissues. Polysaccharide-based drug delivery systems also offer the ability to release drugs in a controlled manner in response to various stimuli, such as the pH, temperature or enzymes, making it possible to deliver drugs at the right time and place [9,10]. Moreover, their use can reduce the frequency of drug administration, minimizing side effects and improving patient comfort [11]. 

Furthermore, PSAs are widely used in the wound-healing process. Polymers such as chitosan, alginate and sodium hyaluronate are used to create dressings that not only protect the wound from infection but also promote tissue regeneration by maintaining a moist environment, promoting angiogenesis and stimulating fibroblast activity [12,13]. Polysaccharides in the field of biosensors are a key element in the design of biosensors, which are capable of detecting various biomolecules and analytes with high sensitivity and specificity [14,15]. As for cell culture, substances such as agar, alginate and hyaluronic acid are commonly used as cell culture media. Due to their hydrophilic properties and ability to form gels, they provide a suitable environment for cell growth and differentiation [16].

**Table 1:** Advantages and disadvantages of selected polysaccharides.

Polysaccharide	Advantages	Disadvantages	References
Chitosan	Nontoxicity Biocompatible Biodegradable Antibacterial properties Antifungal properties	Insoluble in water and most organic solvents, pH < 6	[17,18]
Hyaluronic acid	Bio-functionality Biocompatible Biodegradable Ability to bind water	Poor mechanical properties Rapid degradation via oxidative species and enzymatic degradation High cost	[19,20]
Pullulan	Nontoxicity Biocompatible Biodegradable Antimicrobial activity Anti-inflammatory activity Non-immunogenic activity Excellent flexibility High water-absorbing capability Good solubility Film-forming ability Thermal stability	High cost—three times more expensive than other polysaccharides Low mechanical properties	[21,22]
Arabinoxylan	Antioxidant properties Anticancer properties Prebiotic properties Immunomodulatory properties	High cost	[23,24]
Inulin	Anticancer properties Antioxidant properties Prebiotic properties Regulating blood sugar levels Regulating blood lipid levels Regulating immunity	Poor mechanical properties	[25]
Dextran	Biocompatible Biodegradable Nontoxicity Modifiable functional groups Proven clinical safety	High cost	[26]
Chondroitin sulfate	Nontoxicity Biocompatible Antioxidant effect Anti-angiogenic properties Anti-inflammatory activity	Allergic and immunologic reactions	[27]
Heparin	Anticancer activity Anti-coagulation activity Antiviral activity Anti-angiogenic activity Antiproliferative effect	Heparin-induced thrombocytopenia (HIT)	[28]

The application potential of polysaccharide-based biomaterials is evidenced by the advanced preclinical studies on animal models, which are being conducted with increasing frequency. In the case of class-three medical devices, this step is essential for the material to be directed to human trials. Most of these types of studies are conducted on small animals like mice, rats or rabbits. Far fewer involve studies on dogs, goats or pigs. In the case of pullulan and dextran, these studies are mainly concerned with bone applications [29]. According to the data available via the publicly accessible portal clinicaltrials.gov, pullulan has undergone clinical trials but not strictly in the bone aspect. A total of seven clinical trials have been conducted across China, Japan and the US, among others. Significantly more results have been obtained for pullulan, as many as 286 clinical trials worldwide, 6 of which are directly related to bone tissue (five studies completed, one at the recruitment stage in the UK). Chitosan has been subjected to 141 clinical trials, 8 directly related to bone tissue, with the oldest dating back to 2014. Statistically, the polysaccharide chondroitin sulfate is the most popular of the number of clinical trials on the bone topic. Of the 102 observational studies conducted, nearly 40% involve hard tissue. The least amount of research is carried out with arabinoxylan, only 33 studies, of which only 1 is related to bone. The above data are current as of 13 August 2024.

## 3. Selected Polysaccharides in Bone Tissue Regeneration

### 3.1. Chitosan

**Figure 2:**
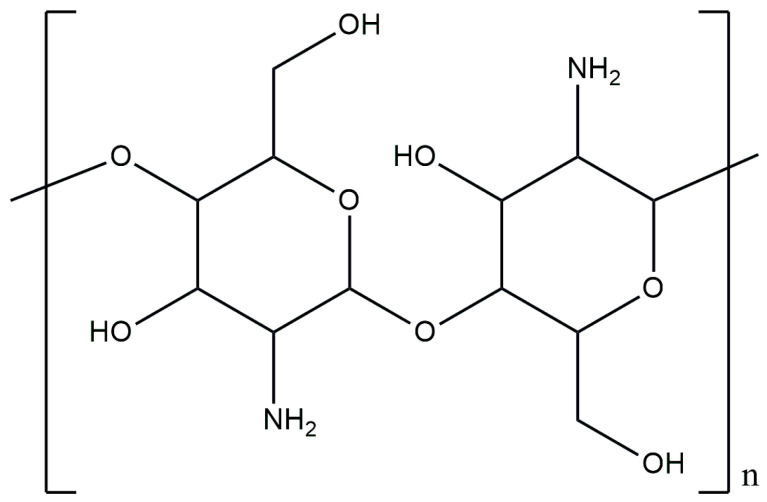
Chemical structure of chitosan.

Chitosan (CS) is a polysaccharide derived mainly from the partial deacetylation of chitin, leading to the formation of a copolymer of N-acetylglucosamine and D-glucosamine, as presented in Figure 2 [30,31]. Depending on the type of primary source of chitosan, which can be crabs, shrimp, or snail shells, the properties of the obtained chitosan such as the molecular weight, degree of deacetylation (DD) and solubility are determined [32,33]. This polysaccharide is perfectly soluble in dilute aqueous solutions of organic acids such as ethanoic acid and methanoic acid [34,35]. Factors that affect the solubility of chitosan are the presence of the D-glucosamine moiety, the molecular weight and the degree of deacetylation [36]. Chitosan, which is one of the key polysaccharides used in biomaterials, is distinguished by such properties as its biocompatibility, biodegradability, nontoxicity and antimicrobial activity [37,38,39]. These characteristics make it widely used in various industrial sectors. Applications of chitosan include the production of dressing materials, implants and drug carriers [40,41,42]. Moreover, this polysaccharide is digested by the human body’s enzymes and aids the wound-healing process, promoting hemostasis and accelerating tissue regeneration [43]. In addition, chitosan is a promising polymer for use as a matrix in biodegradable and natural composite materials, especially in combination with calcium phosphate ceramics [44,45,46]. 

This polysaccharide is widely used in combination with inorganic phases, particularly calcium phosphate ceramics. Most chitosan-based composites are found with hydroxyapatite nanoparticles due to giving the material bioactivity [47,48,49]. Soriente et al. proposed a method to obtain biocomposite scaffolds with high amounts of hydroxyapatite (HAp) nanoparticles by combining sol-gel technology and a freeze-drying process. Biocomposites with higher concentrations of inorganic fillers (60 and 70%) demonstrated an effect on the osteogenic differentiation of human mesenchymal stem cells (hMSCs) toward a mature osteoblast phenotype and were able to prevent, in an in vitro cell culture model, pro-inflammatory events [50]. Moreover, it is possible to obtain a multi-stage bone scaffold with a core–shell porous structure. This core–shell scaffold can be formed using polyvinyl alcohol/polylactic acid (PVA/PLA)-based multilayered braided fibers wrapped in chitosan with hydroxyapatite. The addition of HAp gave the core scaffold a macroscopic pore size of 80–180 μm, which significantly improved the cell viability and provided application potential in bone tissue engineering [51]. Similar properties were demonstrated by bioactive CS/Si/HAp/Ca-GP composites based on chitosan, inorganic silica, hydroxyapatite (HAp reinforced with calcium β-glycerophosphate (Ca-GP), which were additionally characterized by a widely developed pore surface. In addition to good proliferation and cell growth, the composites achieved shear strengths ranging from 0.3 to 10 MPa, and a Young’s modulus from 5.2 to 100 MPa, making them a promising material for filling small bone defects [52]. Another form of ceramic often used in combination with chitosan is tricalcium phosphate (TCP). An interesting method for obtaining flower-shaped CS/CaP microparticles as a carrier for a drug delivery platform was proposed by Luo et al. The results suggest good prolonged release properties of CS/CaP microparticles, and the cumulative release time can exceed 24 h. In addition, the in vitro cytotoxicity test shows the excellent biocompatibility of CS/CaP microparticles, which gives rise to wide application as a DDS, especially in bone regenerative medicine [53]. It is also possible to produce chitosan-based tricalcium phosphate (β-TCP) scaffolds coated with silk fibroin. The resulting scaffolds have porosity of up to 86%, range 60–170 μm and stiffness in the range of 1 to 2 MPa. More importantly, the formed composites with polysaccharide and bioactive ceramics stimulated the material to form apatite crystals in culture with mouse preosteoblast MC3T3 cells, which shows potential for application in hard tissue regeneration [54]. Growth factors (GFs) are very important in the process of bone regeneration. In order to improve bone healing, the brushite–chitosan system was developed to control the release kinetics of VEGF and PDGF. Interestingly, the brushite–chitosan system is able to regulate the rate of release and localization of both growth factors in the bone defect, and thus contributes significantly to bone repair [55]. A rather attractive method for obtaining chitosan-based biomaterials in combination with CaP is 3D printing. Printed scaffolds for hard tissue engineering based on chitosan are obtained in combination with hydroxyapatite [56], tricalcium phosphate [57], and brushite [58].

### 3.2. Hyaluronic Acid

**Figure 3:**
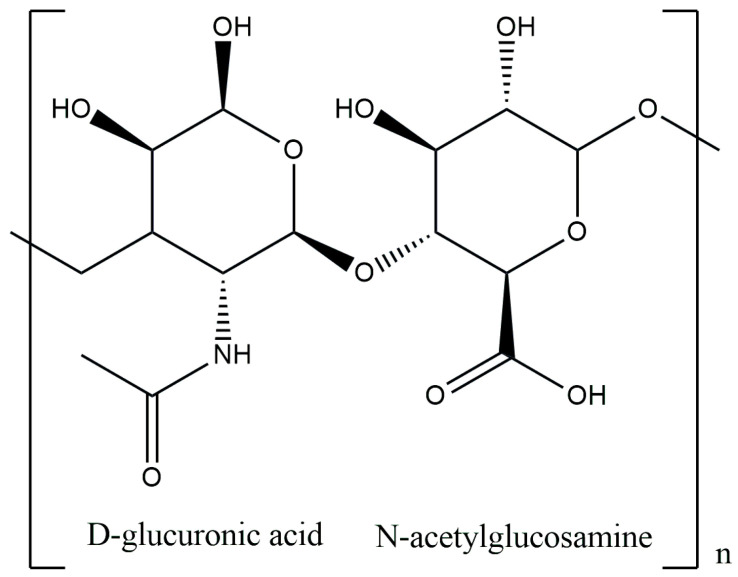
Chemical structure of hyaluronic acid.

Hyaluronic acid (HA), known as hyaluronate, is the main component of the skin ECM, belonging to the anionic group, non-sulfated heteropolysaccharide of glycosaminoglycans [59]. In living organisms, it is found in the vitreous body, joints, umbilical cord, skin or connective tissue, and it is involved in the inflammatory response, angiogenesis as well as the tissue regeneration process. From the chemical point of view, HA is an unbranched polymer, polar in nature (hydrophilic), built from repeating disaccharide units of D-glucuronic acid and N-acetylglucosamine, linked by β-1,3 and β-1,4 glycosidic bonds (Figure 3) [60,61]. In 1934, it was first discovered in the vitreous fluid of the eye, but only in 1964 was it successfully isolated. It has a wide range of molecular weights from 103 to 107 Daltons, thus distinguishing high-molecular-weight HA (HMW-HA) and low-molecular-weight HA (LMW-HA). This affects the final action and properties of this polysaccharide. HMW-HA exhibits angiogenetic activity and protects articular cartilage due to its lubricating properties, while LMW-HA, on the other hand, can induce tumor progression or exhibit pro-inflammatory activity. However, the most important property of HA is its ability to capture 1000 times its water weight [62,63]. Considering its biocompatibility and degradability, it is used in many biomedical applications, like drug encapsulation and delivery platform, cosmetic fillers or skin care [64,65]. 

Naturally, this polysaccharide is also widely used in tissue engineering related to bone tissue regeneration. However, due to its low strength properties, in research, HA is often combined with other polymers such as chitosan (CS), collagen (COL) or gelatin (GE). A combination of HAp, HA, and COL was proposed by Subramaniam et al., developing a composition primarily for cavities resulting from periodontitis but indicating that the material has application potential for bone as well. In vivo analyses and histological staining demonstrated an increased number of osteocytes in cavity sites treated with this implant, confirming activity toward the bone remodeling process [66]. Another approach involves using the same ingredients to obtain porous foam structures in the form of scaffolds on which cells will proliferate. As opposed to the previously presented solution, which was chemically crosslinked, freezing and subsequent lyophilization were proposed [67]. The number of literature reports regarding the blending of these natural polymers with HAp are extensive and demonstrate the great interest in this topic as well as highlight the potential of such a combination [68,69,70,71,72]. GE is a common additive to other hydrogel materials because of its gelling properties [73]. By combining GE/HA/HAp and using a chemical crosslinking method, both 3D-printed scaffolds and composites for bone defect fillings were obtained. In both cases, the biomineralization process of the new layers was observed, confirming the potential of the materials in biomedical applications. However, the bioactivity of the composite could be considered higher, considering the extra modification with bone morphogenic protein (BMP) [74,75]. By modifying the methylacrylation of GE and HA, it is possible to obtain solid composite materials using the UV light photocrosslinking method. However, in order to achieve this, it is necessary to modify the amino group of the GE molecular chain as well as the hydroxyl and carboxyl groups of HA [76]. Instead of GE, PVA is also used as a gelling agent [77].

The other inorganic bioactive ceramic materials combined with HA include TCP. A tiny addition of HA as a gelling agent allowed TCP-based bone cements to be obtained with satisfactory performance. Cements containing 1% and 4% HA were tested. Improvements in the compressive strength were observed with increased amounts of HA as well as enhanced mRNA expression of hBMSCs, i.e., human bone marrow mesenchymal stem cells. Thus, HA is a highly anticipated additive to improve the physicochemical properties and osteoinductivity of cements [78]. In other studies, HA provided a medium for β-TCP deposition in granular form. The polysaccharide is characterized by its disappearance over time, and the ceramic generates osseointegration reactions [79]. Combining them with Ag nanoparticles and corn silk yielded a thermosensitive, injectable hydrogel system that improved osteogenesis. The metallic nanoparticle content further provided antibacterial properties [80]. It is important to emphasize that the nature of polysaccharides allows them to be used as carriers of active substances, like proteins or drugs. HA/COL/β-TCP was modified with tetracycline. Drug release studies demonstrated that more than 90% of the tetracycline was released after 5 days. Additional evaluation of the biocompatibility and promotion of MG-63 cell proliferation highlighted the potential of this material [81]. Other studies assume modification with clindamycin, an antibiotic with broad antibacterial activity. However, bruhite was used instead of TCP. The study highlights the effect of ceramic content on the amount of drug released [82]. An important active factor in bone regeneration is the BMP-2 protein. Scaffolds were developed for local bone defects. The length ratio of new bone formation in the BMP-2-loaded case was significantly higher than that in the only HA/TCP group [83].

### 3.3. Pullulan

**Figure 4:**
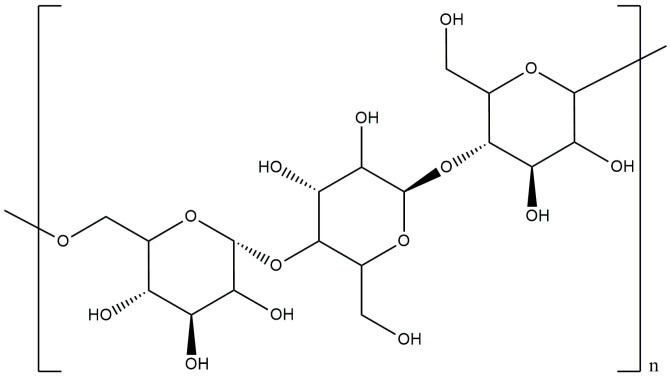
Chemical structure of pullulan.

Pullulan (PUL) is a microbial polysaccharide that is produced aerobically as an amorphous slime from the yeast-like fungus *Aureobasidium pullulans* [84]. The structure of pullulan consists of maltotriose units that are linked together by α-1,6-glycosidic bonds, as presented in Figure 4 [85]. This polysaccharide comes in the form of a white or yellowish-white powder with a pH of 5 to 7. The enzyme that hydrolyzes pullulan is pullulanase, which can be distinguished by two types, i.e., type I and type II. Type I pullulanase interacts with the α-(1,6) glycosidic bond to form the maltotriose unit. Type II pullulanase acts on the α-(1,6) glycosidic and α-(1,4) glycosidic bond to form maltotriose and a mixture of mannose, glucose and maltotriose, respectively [86,87]. Due to its unique structure made of repeating maltotriose units, pullulan has high structural flexibility, high mechanical strength, and exhibits membrane-forming properties [88]. In addition, pullulan has functional properties like film-forming ability, enzyme-mediated degradation ability, and adhesiveness [89,90]. The viscosity of a pullulan solution depends on the molecular weight. The viscosity of a pullulan solution remains constant despite changes in the temperature, pH and the presence of most metal ions, including sodium chloride [91,92]. PUL is biocompatible, biodegradable, non-mutagenic, non-toxic, non-carcinogenic, non-immunogenic and has been declared safe by the FDA in the United States and is generally recognized as safe (GRAS) [91,93].

**Figure 5:**
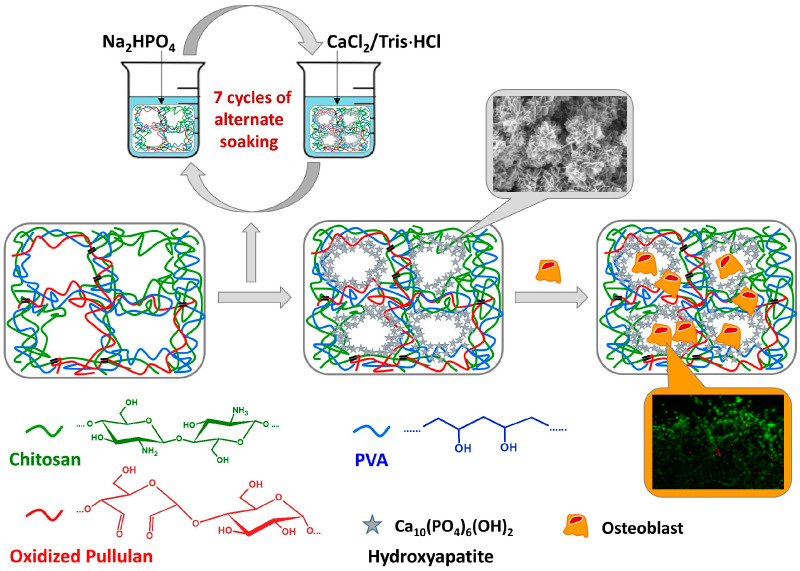
Scheme for obtaining composites based on pullulan, chitosan, and PVA with hydroxyapatite for bone tissue engineering. Adapted from Ref. [96], MDPI, 2023.

Due to its properties, pullulan is often used to create hydrogels that can be modified with ceramics. The most common ceramics used for this purpose are calcium phosphate ceramics such as hydroxyapatite. Gels based on hyaluronic acid and pullulan were modified with (3-glycidyloxypropyl)trimethoxysilane (GPTMS) and incorporated into spheres of biomimetic hydroxyapatite (BHa). The addition of BHa increased the storage modulus and viscosity and provided greater stability in terms of the mechanical behavior. In addition, the swelling ratio and water content decreased, but by the addition of BHa, the alginate–pullulan gels were resistant to rapid enzymatic degradation, which significantly affected the survival and proliferation of L-929 fibroblast cells, especially around the biomimetic hydroxyapatite spheres [94]. Due to the poor mechanical properties of hydrogels in bone tissue engineering applications, reinforcement with nanocrystalline hydroxyapatite (nHAp) (5% wt. nHAp in hydrogel) and poly(3-hydroxybutyrate) (PHB) fibers (3% wt. fibers in hydrogel) containing nHAp (3% wt. nHAp in fibers) was proposed. The addition of fillers resulted in an improvement of the elastic moduli by 10 times [95]. Moreover, Pelin et al. developed composite hydrogels based on oxidized pullulan, chitosan and PVA that can be modified with calcium phosphates, according to the scheme presented in Figure 5. In vitro bioassays revealed that mineralized hydrogels were more conducive to the proliferation of MG-63 osteoblast-like cells compared to non-mineralized hydrogels [96]. PUL is also used as a modifier of bone cements. One such solution is the modification of bone cement based on dicalcium phosphate dihydrate (DCPD) with this polysaccharide. The results indicate that the presence of pullulan was associated with an increase in compressive strength up to 20.4 MPa and an increase in the cement setting time. In addition, the arrangement of DCPD with pullulan counteracted the collapse by the interfacial bond between PUL and DCPD that occurred, suggesting that this composite has potential for application in the field of bone regenerative medicine [97]. Moreover, it is often processed using electrospinning to produce 3D fibrous scaffolds in bone tissue engineering applications [98,99]. Pullulan nanofibers were developed using cricket powder as an innovative ingredient due to its high chitin and chitosan content. Hydroxyapatite was also added to enhance the biocompatibility and ability to interact with host tissues. The safety and efficacy of these scaffolds were evaluated in vivo on a mouse model of incision and burn. The results showed that the foamed scaffolds containing cricket powder not only had better structural and mechanical integrity but also higher cytocompatibility compared to various cell lines [100]. Fibrous 3D networks have been produced by electrospinning pullulan with cellulose acetate (CA) in various ratios, and then crosslinking the scaffolds with trisodium trimethaphosphate (STMP). The scaffolds also promoted the formation of apatite-like structures after incubation in simulated body fluid (SBF) and facilitated Saos-2 cell adhesion and proliferation, indicating the potential for osteoblastic differentiation [101]. In comparison, another study used diatom shells (DSs) to engineer bone tissue, combining them with biopolymers such as poly(hydroxybutyrate-co-hydroxyvalerate)/poly(ε-caprolactone) (PHBV/PCL) and PUL fibers to create multifunctional 3D scaffolds. These scaffolds exhibited controlled antibiotic release, improved mechanical properties, and better cell viability and osteocompatibility. Notably, the DS-containing scaffolds also promoted the formation of an apatite layer, which is crucial for bone integration [102]. Moreover, Popescu et al. implanted scaffolds based on alginate, pullulan, and bioactive glass–ceramic with 0.5 and 1.5 mol% copper oxide into rat models to evaluate their ability to heal bone defects. Observations using MRI, imaging scans, and histological evaluation showed progressive bone healing within 5 weeks, with more new bone tissue forming around the scaffolds. The greatest bone regeneration (37%) was observed with an implant containing 1.5 mol% CuO, while the bone regeneration achieved by the scaffold with 0.5 mol% CuO was comparable to that of an alginate–pullulan–β-calcium phosphate/hydroxyapatite composite implant [103].

### 3.4. Arabinoxylan

**Figure 6:**
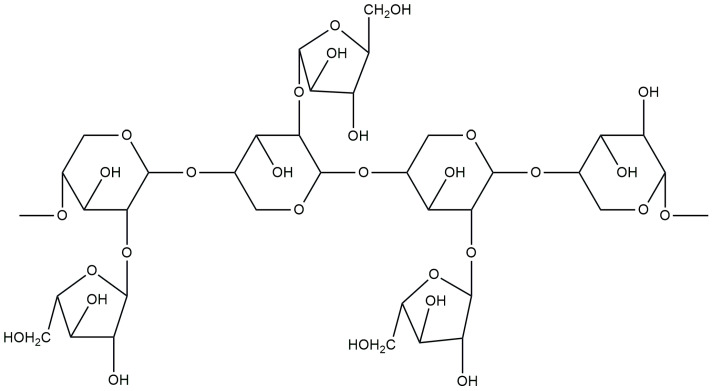
Chemical structure of arabinoxylan.

Arabinoxylans (AXs) are commonly present in the cell walls of many plants, particularly in cereal grains such as wheat, rye, oats, barley and corn. They are the main component of dietary fiber in these grains [104]. AX consists of β-(1,4)-linked D-xylopyranosyl residues to which α-L-arabinofuranose units are attached as side chains, as shown in Figure 6 [105,106]. This polysaccharide is characterized by its natural hydrophilicity and significant ability to form hydrogen bonds. Nevertheless, its hydrophilic and neutral nature may limit its reactivity and potential applications in various fields. Therefore, there is a need to functionalize AX with the aim of introducing new functional groups and increasing its reactivity, which will enable the wider application of this polysaccharide in industry and regenerative medicine [107]. In addition, the antioxidant and anticancer properties of these polysaccharides make them attractive candidates for cancer therapies, especially in the treatment of colon cancer [23].

Due to their biocompatibility and ability to form gels, arabinoxylans are being intensively studied as components of biomaterials used in regenerative medicine. These properties make them ideal for use as matrices to promote cell growth and as drug carriers for controlled release of active ingredients. An interesting solution is to obtain arabinoxylan from the seed husks of *Plantago ovata* and oxidize it with periodate, leading to the formation of oxidized AX (OAX) with novel properties. A solution-casting method was used to prepare biocompatible mixtures of arabinoxylan with chitosan (AX/CS) in different weight ratios. Studies on the release of the model drug, diclofenac sodium (DC), showed that the OAX/CS blends provided prolonged drug release, with the best results obtained for the OAX/CS blend (1:2), highlighting the potential of oxidized blends in controlled drug delivery systems [107]. Moreover, it is possible to create composite hydrogels based on arabinoxyalan, chitosan and reduced graphene oxide (rGO), which were obtained by crosslinking with tetraethyl orthosilicate (TEOS). In addition, the obtained biomaterials were modified with the antimicrobial drug silver sulfadiazine. The release profile showed a gradual release of the active substance, while tests on MC3T3-E1 cells showed good adhesion, as well as a cell viability of 91% [108]. Similar composites were obtained by Al-Arjan et al. based on arabinoxylan, apple pectin, which were modified with the phosphate calcium ceramics nanohydroxyapatite (nHAp) and graphene oxide (GO), as a potential application in bone regenerative medicine. It was noted that with increasing amounts of graphene oxide, the porosity and mechanical properties of the nanocomposites are regulated [109]. Biocomposite scaffolds for bone tissue engineering applications can also include a matrix based on arabinoxylan, polyvinyl alcohol and a non-organic phase of nanohydroxyapatite and graphene oxide. The obtained biomaterials showed improved antimicrobial activity, as well as no effect of blood coagulation [110]. To improve the antimicrobial activity, polymeric nanocomposite scaffolds based on arabinoxylan-acrylic acid, nano-hydroxyapatite (nHAp), nano-aluminum oxide (nAl_2_O_3_) and graphene oxide (GO) were coated with silver (Ag) nanoparticles. The study showed that the Ag-coated polymer nanocomposite scaffolds had excellent antibacterial properties and a better microstructure. Along with the amount of GO, the compressive strength (18.89 MPa) and Young’s modulus (198.61 MPa) improved, providing application opportunities in hard tissue engineering [111]. In bone tissue engineering, an interesting thread is the combination of arabinoxylan with nano-hydroxyapatite (nHAp) and titanium dioxide (TiO_2_). Studies demonstrate that such scaffolds have good biocompatibility and allow the normal growth of MC3T3-E1 cells [112]. Good biocompatibility properties promote the integration of the implant into the bone, which in turn leads to successful osteointegration.

### 3.5. Inulin

**Figure 7:**
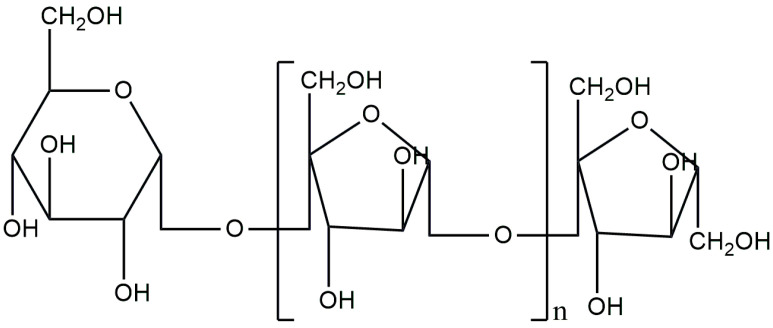
Chemical structure of inulin.

Inulin (INL) is a natural fructan polysaccharide found abundantly in nature. Depending on the chain length, it is also classified as an oligosaccharide. It can be extracted from Jerusalem artichoke, chicory, banana, onion or wheat [113,114]. INL is characterized by its biodegradability and is relatively soluble in water. In 2002, it was approved by the FDA as GRAS. In the field of tissue engineering, INL is readily used as a drug carrier, scaffold or component of composite biomaterials [115,116]. The chemical structure of INL (Figure 7) consists of β-d-fructosyl subgroups linked to each other by (2 → 1) glycosidic bonds [117].

Despite the many features of INL that make it a good material for biomedical use, there are not many reports of its applications in the aspect of bone tissue. Most of these relate to drug delivery systems [118]. Like the heparin described below, INL can be used to functionalize other materials. HAp powder was suspended in a solution of a derivative of this polysaccharide for modification. The effect of the amount of carboxymethyl INL on the morphology of the powder was demonstrated. Such a conjugate was used as a carrier for ibuprofen [119]. Another solution proposes using INL as a component of a filament for printing bone defects. For this purpose, it was first combined with PLA and then with PCL. Both PLA and PCL are used in the bioprinting industry. The goal of related studies was to determine the effect of INL as a modifier. Ultimately, it was demonstrated that it exhibited high homogeneity. Furthermore, better surface wettability (hydrophilicity) and swelling ability, and a higher in vitro biodegradation rate, were observed compared to only PCL [120]. 

### 3.6. Dextran

**Figure 8:**
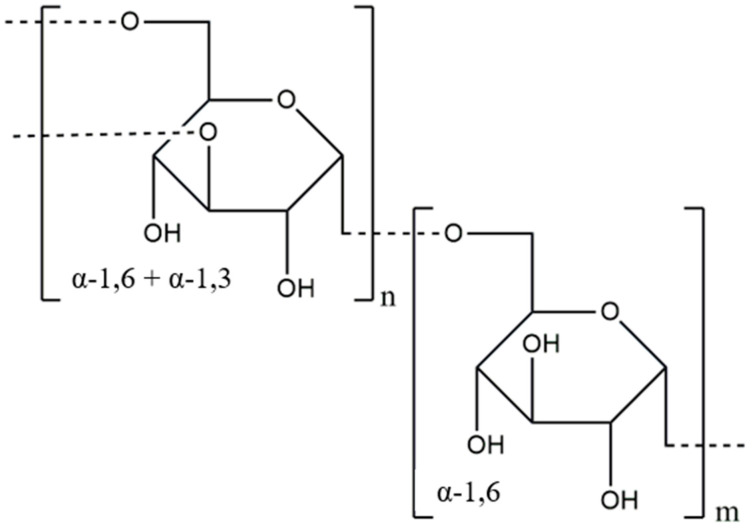
Chemical structure of dextran.

Dextran (DEX) is a polysaccharide of natural origin, mainly composed of D-glucose monomers linked by α-(1 → 6) bonds, as shown in Figure 8. Depending on the production method used, other types of bonds, such as α-(1 → 2), α-(1 → 3) and α-(1 → 4), may also appear in the structure of dextran [26,121]. Dextrans are synthesized by the extracellular enzyme dextransucrase, which is secreted by lactic acid bacteria (LAB). This enzyme catalyzes the hydrolysis of sucrose and the polymerization of the released glucosyl units to form dextran [122]. This polymer exhibits hydrophilicity and biocompatibility, making it a potential material for use in regenerative medicine [123]. Clinical-grade dextran with a molecular weight of 40, 60, or 70 kDa in aqueous solutions can act as a blood plasma substitute [124,125]. Interestingly, dextran with a molecular weight of 70 kDa is on the World Health Organization’s (WHO’s) model list of essential medicines [126].

**Figure 9:**
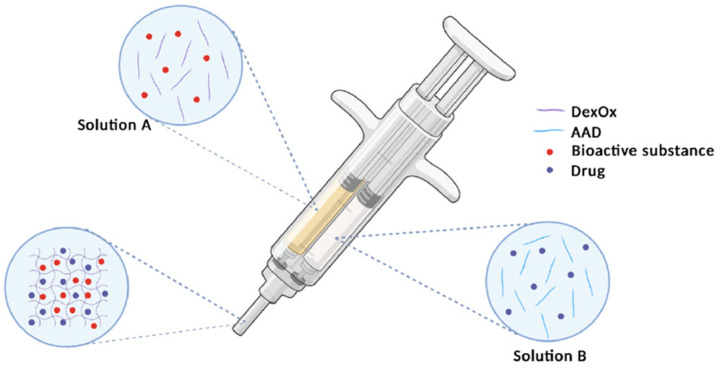
Schematic representation of injectable dextran-based hydrogels with calcium phosphate ceramic and the antibiotic vancomycin. Adapted from [129], MDPI, 2023.

Dextran-containing composites show significant potential in regenerative medicine, thanks to their biocompatible and biodegradable properties, which promote tissue regeneration and wound healing. In order to combine good mechanical and biological properties, a scaffold based on acrylamide (PAAm) and dextran urethacrylate (Dex-U) was developed, along with modification with hydroxyapatite nanocrystals. The obtained biocomposites exhibited high compressive strength of about 6.5 MPa and stimulated osteogenic differentiation effectively. Moreover, in vivo studies on a rabbit model of a femoral condyle defect demonstrated the regeneration of highly mineralized bone tissue and osseointegration ability [127]. A very important aspect in terms of hard tissue regeneration is the non-removable coating. Shi et al. attempted to create self-repairing coatings based on oxidized dextran (OD), 3-aminopropyltriethoxysilane (APTES) and nano-hydroxyapatite (nHAp) for magnesium scaffolds. The researchers reported that the orpacated OD-MHA/Mg scaffold promotes bone repair by enhancing the osteogenesis gene and protein expression and healing properties in infectious bone damage [128]. Another solution to the problem of bone infection is the development of an injectable hydrogel based on dextran polysaccharide together with ceramics, i.e., β-TCP and nHAp, as shown in Figure 9. In addition to incorporating the bioactive calcium phosphate ceramic into the structure, the researchers incorporated the antibiotic vancomycin into the hydrogel matrix to impart antimicrobial properties. Studies of the release of the active ingredient from the dextran-based biomaterial show a release of 50 to 80% during the initial incubation time [129]. An interesting report presented by Chen et al. is the combination of a composite scaffold based on a hydrogel matrix of oxidized dextran/GE and magnesium–calcium–phosphate cement, which consists of magnesium oxide (MgO), calcium dihydrogen phosphate and β-tricalcium phosphate. The polymer phase forms a 3D lattice structure, while the β-TCP-based cement improves the mechanical properties of the biocomposites. The interaction between the hydrogel and MgO effectively regulates the nucleation sites, and calcium and magnesium ions provide suitable conditions for the growth of apatite layers in in vitro tests [130]. Other studies investigated dextran-based nanocomposite scaffolds with dicalcium phosphate nanoparticles (DCPs) and carboxymethylcellulose formed by freeze-drying. In addition, the biomaterial was modified with the anticancer drug paclitaxel. Scaffolds with higher amounts of DCP and drug had low cytotoxicity compared to the same materials without the active substance [131]. Moreover, the dextran hydrogels can be modified with bioactive gold nanoparticle ceramic nBGC with a composition of 64% SiO_2_, 31% CaO, and 5% P_2_O_5_. During incubation in simulated body fluid, apatite layers were formed on the surface of the nanocomposites and the cross-section. Human osteosarcoma cells (SaOS-2) were able to adhere and proliferate in dextran hydrogels containing bioactive glass nanoparticles [132]. 

### 3.7. Chondroitin Sulfate

**Figure 10:**
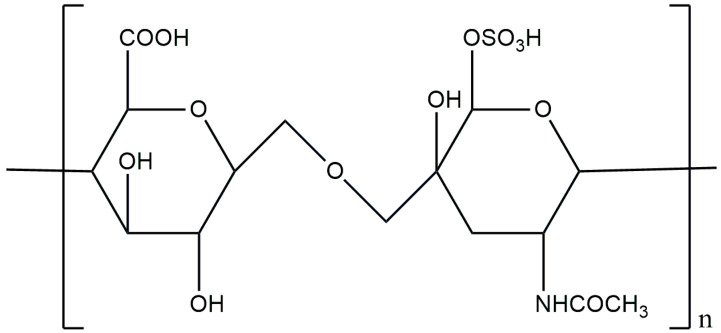
Chemical structure of chondroitin sulfate.

Chondroitin sulfate (ChS) is a natural macromolecule found in the ECM that belongs to the glycosaminoglycan (GAG) class. The structure of ChS consists of two repeating disaccharide units of N-acetylgalactosamine and D-glucuronic acid linked by β-1,3 and β-1,4 bonds [133,134]. Its structure is presented in Figure 10. Naturally occurring ChS is found in the cartilage and other tissues of the body. On top of its biocompatibility, it has a number of useful biological properties [135]. It exhibits antioxidant, anti-inflammatory, immunomodulatory and anticoagulant effects, and it can be used to treat cardiovascular pathologies. Commercially, ChS of natural origin isolated from selected animal tissues is most commonly used. The largest amounts are found in pigs, particularly in their ears, nasal septum and skin, but also in chicken cartilage or bovine trachea. Alternative marine sources of ChS include squid or mollusks [136,137].

Using the phenomenon of photocrosslinking, an in situ injectable composite hydrogel based on chondroitin sulfate and hydroxyapatite was developed for cell encapsulation. Mesenchymal stem cells were encapsulated in this carrier and the cell viability as well as osteogenic differentiation were evaluated. Microscopic observation confirmed that the cells remained viable in the material, which confirms that the system can be used in bone tissue engineering [138]. An interesting use of ChS is proposed by Xiufeng Xiao et al. After combining ChS/HAp, they demonstrated that chemical interactions between ceramic crystals and pre-organized ChS functional groups lead to the nucleation and growth of a new HAp crystals. They also proved that the concentration of ChS significantly affects their morphology; at low concentrations of ChS, fiber-like crystals are formed, and at higher concentrations, flake-like crystals appear [139]. The possibility of further modifying such composites with immunoglobulin (IgG), human IgG (hIgG) and denosumab (Dmab) proteins with and without Zn ions was also described. It was shown that the addition of ions increased the release of antibodies from HAp/ChS in saline in phosphate buffer. In the case of composites containing proteins without Zn, their release was also observed, but to a lesser extent [140]. Although ChS has a number of unique properties, in order to obtain the ideal bone- or cartilage-like material, this polysaccharide is often combined with COL. The ChS/COL/HAp composite, which partially mimicked the composition of cartilage, showed strengths in the range of 35–50 MPa, which is close to the strength of natural cartilage [141]. Another ChS/COL/HAp combination in the form of scaffolds, exhibiting porosity in excess of 90%, demonstrated potential for future bone tissue engineering. Although COL itself is present in bone tissue and generates osteogenesis, it was indicated that ChS/COL/HAp composites showed better cell proliferation and adhesion than COL alone [142,143]. A bone cement composition was also developed based on these three components. In vivo studies on a sheep tibia model, the direct effect of ChS on remodeling and new bone formation around the implant was evaluated. X-ray examination demonstrated a significantly earlier callus response around the HA/Col/CS implants compared to the material without ChS. In addition, the amount of newly formed apatite layers at the endpoint of the experiment was significantly greater around the cements with ChS in both the CT and histomorphometric analysis [144]. Similar conclusions were obtained after changing the cement form to a coating applied to metallic implants composed of Ti [145].

Considering the possibilities of materials engineering, as well as the properties of other polysaccharides, ChS was also combined with the previously described CS [146] or HA. Due to their synergistic action, as well as their potential for osteogenesis, the former demonstrated excellent mechanical, biocompatible and bioactive properties [147].

### 3.8. Heparin

**Figure 11:**
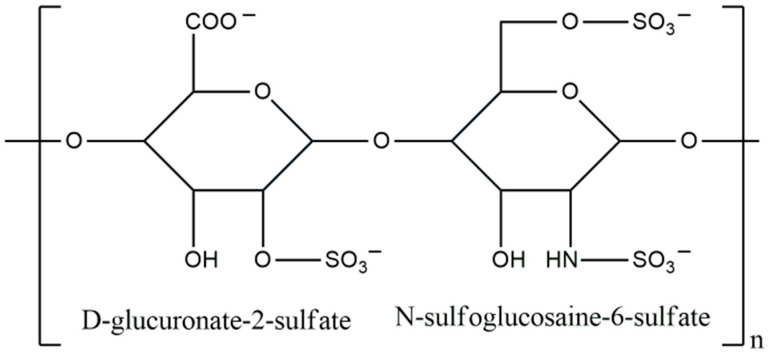
Chemical structure of heparin.

Heparin (HE) is a linear polysaccharide that is composed of repeating units of uronic acid and glucosamine residues. It was first discovered in 1916 and since 1935 has been used clinically as an anticoagulant [148,149]. Its structure is presented in Figure 11. Naturally, HE is produced only in mast cells, where it is cleaved from serglycin (a core protein) at the end of synthesis. For commercial purposes, it is most often isolated from the bovine lung or porcine intestine, and it has an average molecular weight of around 15 kDa [150,151]. However, it is worth bearing in mind that HE produced from different animal species and organs may differ slightly in terms of the structural features, MW, or physicochemical properties [152]. 

A very frequent use of HE is not as a composite phase but rather as a component for functionalizing ceramics or polymers. An example is the modification of HAp, where HE in powder form was added at different weight ratios during the synthesis of ceramics. The functionalized HE/HAp conjugate formed in this way exhibited lower crystallinity and a smaller crystal size compared to pure ceramics; however, they demonstrated better antithrombotic properties. Thus, such a material minimizes the risk of clot formation in the area of the implanted biomaterial [153]. Other studies report that the addition of this polysaccharide during the synthesis of ceramics makes it possible to obtain a material with controlled morphology [154]. It is also possible to pre-modify HE prior to HAp functionalization. Such a solution was proposed by Sung Eun Kim et al., creating a conjugate of HE with dopamine. To combine these two, 1-ethyl-3-(3-dimethylaminopropyl)-carbodiimide (EDAC) and N-hydroxysuccinimide (NHS) were used. Such a conjugate was then mixed with ceramic, and additionally, at the end, with lactoferrin, the release of which was determined over time. It was shown that the system thus formed was able to increase the osteogenic differentiation of rabbit adipose tissue-derived stem cells [155]. In another study with HE, NHS and EDAC were also used, albeit as a crosslinker for the HE/COL/HAp-based system. It was proposed to obtain porous ceramic-based scaffolds that were coated with COL and HE to form a layered composite. The idea behind this solution was the controlled and prolonged delivery of BMP-2 protein. Comparing the scaffolds with polysaccharide to materials without it, it was observed that the presence of HE significantly reduces the characteristic initial phase of release; at the same time, the release profile of BMP-2 could be maintained for 7 days in vitro in a controlled manner [156]. Considering the fact that heparin binds to angiogenic growth factors, affecting the process of new blood vessel formation, an HE/CS/HAp composite was developed. It was confirmed that low concentrations of HE actually showed a positive effect, even at an amount of less than 30 μg per scaffold, indicating a significant increase in blood vessels [157]. In situ hydrogels based on HE were also described. The aforementioned CS was combined with ceramics and HE to form an injectable gel for bone defects. The resulting hydrogel networks had a flat morphology similar to bone with hierarchical porosity, which consists of large pores (50–500 µm) with intersecting walls of micropores (1–10 µm). This system of open pores allows blood vessels to grow in, which minimizes the risk of displacement of the biomaterial [158]. A coating was also developed based on this composition for covering implants. In this case, CS was functionalized with HE, which was then combined with HAp. The purpose of the modification was to improve the compatibility with blood [159]. For a similar reason, coating compositions have been developed for dental applications. Coatings based on pure HAp are often used in dentistry, including commercially. However, an HE/HAp coating on titanium (Ti) has the additional benefit of preventing blood clotting. Comparing pure Ti, HAp coating and HAp/HE, it was demonstrated that the cell viability was significantly lower on the bare metal surface, while the latter material exhibited relatively better performance than ceramics, while the cell morphology remained unchanged [160].

## 4. Conclusions and Future Challenges

Polysaccharides are a broad group of compounds of natural origin that can be used in medicine due to their unique properties. Moreover, the possibility of functionalizing both polysaccharides and other materials with these compounds, such as heparin, creates great opportunities for the development of smart carriers, multifunctional scaffolds or fillers. The above review presents an overview of selected polysaccharides as biomaterials for bone tissue. It can be assumed that they are non-toxic, and their structural similarity to GAG is responsible for their biocompatibility, which is an essential ingredient of the ECM. The growing interest in biodegradable materials is leading to increased research into polysaccharide-based composites, which can replace conventional plastics in many applications, providing a reduction in environmental pollution. Techniques for combining different groups of materials make it possible to create composites with better mechanical, thermal and biological properties, which will broaden their range of applications. Polysaccharide composite systems will find wide application in medicine, especially in areas such as drug delivery, tissue engineering and the creation of biodegradable implants. Work on the controlled release of active substances and tissue regeneration will be key to future advances. This article summarizes the knowledge developed over the past few years on polysaccharide-based composite materials, covering their properties, synthesis methods and potential applications. However, there are relatively few articles in the scientific literature in this field, indicating the need for further research. Nevertheless, polysaccharide-based composite materials may be an interesting subject for researchers to develop.

## Data Availability

No new data were created or analyzed in this study.

## Abbreviations

Abbreviation	Meaning
AX	arabinoxylan
BHa	biomimetic hydroxyapatite
BMP	bone morphogenic protein
BTE	bone tissue engineering
CAGR	compound annual growth rate
CaP	calcium phosphate ceramic
ChS	chondroitin sulfate
COL	collagen
CS	chitosan
DCPD	dicalcium phosphate dihydrate
DD	degree of deacetylation
DDS	drug delivery system
DEX	dextran
DS	diatom shell
ECM	extracellular matrix
FDA	Food and Drug Administration
GAG	glycosaminoglycans
GE	gelatin
GF	growth factors
GO	graphene oxide
GRAS	generally recognized as safe
HA	hyaluronic acid
HAp	hydroxyapatite
HE	heparin
HIT	heparin-induced thrombocytopenia
hMSCs	human mesenchymal stem cells
HMW-HA	high-molecular-weight hyaluronic acid
INL	inulin
LMW-HA	low-molecular-weight hyaluronic acid
nHAp	nanohydroxyapatite
PCL	poly(ε-caprolactone)
PDGF	platelet-derived growth factor
PHB	poly(3-hydroxybutyrate)
PHBV	poly(hydroxybutyrate-co-hydroxyvalerate)
PLA	polylactic acid
PSAs	polysaccharides
PUL	pullulan
PVA	polyvinyl alcohol
SBF	simulated body fluid
VEGF	vascular endothelial growth factor
WHO	World Health Organization
β-TCP	tricalcium phosphate
